# High-dose thiamine prevents brain lesions and prolongs survival of *Slc19a3*-deficient mice

**DOI:** 10.1371/journal.pone.0180279

**Published:** 2017-06-30

**Authors:** Kaoru Suzuki, Kenichiro Yamada, Yayoi Fukuhara, Ai Tsuji, Katsumi Shibata, Nobuaki Wakamatsu

**Affiliations:** 1 Department of Genetics, Institute for Developmental Research, Aichi Human Service Center, Kasugai, Aichi, Japan; 2 Department of Nutrition, School of Human Cultures, The University of Shiga Prefecture, Hikone, Shiga, Japan; Johns Hopkins University School of Medicine, UNITED STATES

## Abstract

SLC19A3 deficiency, also called thiamine metabolism dysfunction syndrome-2 (THMD2; OMIM 607483), is an autosomal recessive neurodegenerative disorder caused by mutations in *SLC19A3*, the gene encoding thiamine transporter 2. To investigate the molecular mechanisms of neurodegeneration in SLC19A3 deficiency and whether administration of high-dose thiamine prevents neurodegeneration, we generated homozygous Slc19a3 E314Q knock-in (KI) mice harboring the mutation corresponding to the human SLC19A3 E320Q, which is associated with the severe form of THMD2. Homozygous KI mice and previously reported homozygous *Slc19a3* knock-out (KO) mice fed a thiamine-restricted diet (thiamine: 0.60 mg/100 g food) died within 30 and 12 days, respectively, with dramatically decreased thiamine concentration in the blood and brain, acute neurodegeneration, and astrogliosis in the submedial nucleus of the thalamus and ventral anterior-lateral complex of the thalamus. These findings may bear some features of thiamine-deficient mice generated by pyrithiamine injection and a thiamine-deficient diet, suggesting that the primary cause of THMD2 could be thiamine pyrophosphate (TPP) deficiency. Next, we analyzed the therapeutic effects of high-dose thiamine treatment. When the diet was reverted to a conventional diet (thiamine: 1.71 mg/100 g food) after thiamine restriction, all homozygous KO mice died. In contrast, when the diet was changed to a high-thiamine diet (thiamine: 8.50 mg/100 g food) after thiamine restriction, more than half of homozygous KO mice survived, without progression of brain lesions. Unexpectedly, when the high-thiamine diet of recovered mice was reverted to a conventional diet, some homozygous KO mice died. These results showed that acute neurodegeneration caused by thiamine deficiency is preventable in most parts, and prompt high-dose thiamine administration is critical for the treatment of THMD2. However, reduction of thiamine should be performed carefully to prevent recurrence after recovery of the disease.

## Introduction

Thiamine (also known as vitamin B_1_) is an essential water-soluble vitamin. The body cannot produce thiamine and can only store approximately 30 mg of it in skeletal muscles, brain, heart, liver, and kidneys [1]. Therefore, adult men and women require continuous dietary intake of approximately 1–1.2 mg of thiamine per day [2]. Thiamine is transported into cells mainly by two thiamine transporters (SLC19A2 and SLC19A3) [3, 4]. SLC19A2 is expressed in skeletal muscles and systemic tissues, whereas SLC19A3 is expressed predominantly in the upper intestine and the duodenum [5, 6]. Thus, thiamine is absorbed mainly at the duodenum by SLC19A3 and then transported into tissues and cells by SLC19A2 and SLC19A3. SLC19A3 has a high specificity for thiamine with a Km of 25 nM, whereas SLC19A2 has relatively low specificity for thiamine with a Km of 2.5 μM [5]. Thiamine is converted into the cofactor form of thiamine pyrophosphate (TPP) by a cellular enzyme, thiamine pyrophosphokinase (TPK; EC 2.7.6.2) [7, 8]. TPP is incorporated into four known mammalian enzymes in cellular metabolism: transketolase, involved in the pentose phosphate pathway; pyruvate dehydrogenase and α-ketoglutarate dehydrogenase, associated with the tricarboxylic acid (TCA) cycle; and branched chain α-keto acid dehydrogenase complex, involved in the catabolism of the three branched-chain amino acids (leucine, isoleucine, and valine) [9]. Thus, thiamine is critically important as a cofactor of enzymes associated with ATP generation at mitochondria via the TCA cycle.

Pyrithiamine is a thiamine antagonist that inhibits the synthesis of TPP from thiamine [10] and accumulates in the brain [11]. Experimental mouse models of acute thiamine-deficient encephalopathy have been generated by intraperitoneal injection of pyrithiamine and a thiamine-deficient (TD) diet [12]. These TD mice (or rats) have diminished levels of thiamine-dependent enzymes and altered cerebral energy metabolism, lactic acidosis, abnormalities in oxidative processes, brain edema, selective neuron loss, blood–brain barrier abnormalities, glutamate-mediated excitotoxicity, and astrocyte dysfunction at the vulnerable regions of the thalamus; they have been used to study the pathogenesis of Wernicke encephalopathy [12–16].

Biotin-responsive basal ganglia disease (BBGD), or thiamine-responsive encephalopathy, has been recently named thiamine metabolism dysfunction syndrome-2 (THMD2; OMIM 607483). THMD2 is a neurodegenerative disorder caused by mutations in *SLC19A3*, the gene encoding human thiamine transporter 2 on chromosome 2q36 [17, 18]. In 1998, Ozand *et al*. reported 10 patients with basal ganglia disease characterized by childhood-onset subacute encephalopathy, extrapyramidal symptoms, and quadriparesis with brain MRI lesions of the bilateral caudate nucleus and putamen [17]. These symptoms disappeared after high-dose biotin administration (5–10 mg/kg/day), and the disease was named BBGD. Molecular genetic analysis of these patients revealed that BBGD is caused by two types of missense mutations (G23V and T422A) of *SLC19A3*. Accumulation of subsequent patients with *SLC19A3* mutations revealed that the clinical features and age of onset of THMD2 are quite variable: they range from the most severe forms of neonate or infant onset Leigh-like syndrome [19–21] and childhood onset basal ganglia disease [18, 22], to second decade onset Wernicke’s like encephalopathy characterized by epilepsy, ataxia, nystagmus, ophtalmoplegia, and MRI lesions of the medial thalamus and periaqueductal grey matter are typically affected [23]. The natural course of THMD2 is invariably progressive if the patients are not treated with high-dose thiamine and/or biotin, and may lead to death. These broad clinical features depend mostly on the types of *SLC19A3* mutations (i.e., the patient’s genotype), but the amount of thiamine intake from milk or food could also be associated with disease onset and progression.

We previously reported four Japanese patients presenting with epileptic spasms in early infancy (2.5 months after birth), severe psychomotor retardation, and characteristic brain MRI findings of progressive brain atrophy and bilateral thalami and basal ganglia lesions caused by a homozygous mutation (c.958G>C [p.E320Q]) in *SLC19A3* [19]. However, there is no report on how neurodegeneration develops in THMD2 over time and how the symptoms recover after high-dose thiamine administration using a mouse model of disease. To address these questions, we generated the Slc19a3 knock-in (KI) (E314Q) mice harboring the mutation corresponding to the human SLC19A3 E320Q. In this report, we performed biochemical and immunohistochemical studies of Slc19a3 KI mice and previously reported Slc19a3 knock-out (KO) mice fed with thiamine-restricted diet, and analyzed the effect of high-thiamine treatment on these mice after thiamine restriction.

## Materials and methods

### Bioethics approval

All study protocols, including animal housing, were reviewed and approved by the Animal Experimentation Committee of the Institute for Developmental Research, Aichi Human Service Centre (Permit number: M-13) in accordance with the Guidelines for Animal Experimentation. All efforts were made to minimize the number of animals used and their suffering.

### Mouse breeding and husbandry

Slc19a3 E314Q KI mice were generated by homologous recombination (S1 Fig). *Slc19a3* KO mice were obtained from the Jackson Laboratory (Slc19a3^tm1Said^) [24]. Both strains of mice were bred with their wild-type C57BL/6J counterparts and maintained as heterozygous *Slc19a3* KI or *Slc19a3* KO in a C57BL/6J background. Mice were maintained under standard laboratory conditions (24 ± 2^°^C room temperature, 60 ± 10 relative humidity) and 12-h light (07.00 to 19.00 h)/12-h dark cycle (19.00 to 07.00 h) with *ad libitum* access to food and water. Mice were housed in groups of 2–5 per cage and were identified by ear notches. Since there are both male and female patients with TMHD2 [17, 25], we analyzed both male and female mice in this study.

### Conventional, thiamine-restricted, and high-thiamine–containing food for mice

Mice were maintained routinely with conventional diet (CLEA Rodent Diet CE-2, CLEA), which has a thiamine concentration (thiamine hydrochloride, MW = 337.3) of 1.71 mg/100 g food. We prepared two types of thiamine-restricted food based on “purified diets for laboratory rodents” (AIN-93M) (Oriental BioService Inc.) [26], in which thiamine concentration was 0.60 mg/100 g food (35% thiamine of conventional food) or 0.27 mg/100 g food (16% thiamine of conventional food). A high-thiamine–containing food was also prepared from AIN-93M, in which thiamine concentration was five times that of CE-2 (thiamine: 8.50 mg/100 g food). Thiamine concentration was determined at Japan Food Research Laboratories.

### Study design

Littermates were generated by breeding heterozygous male mice with their female counterparts. PCR-based genotypic analysis of pups was performed at 4 weeks of age. Pups were maintained with their mother on a conventional diet until 5–6 weeks of age (i.e., just until they were weaned from milk), after which they were randomly placed in pairs of cages, each containing 2–3 mice. The onset of THMD2 in human patients occurs usually during infancy or childhood. Mice of corresponding age would be younger than the 5–6-week-old mice used in this study. However, since it is difficult to reduce thiamine intake by lactating mice, we started feeding the thiamine-restricted diet to the mice at 5–6 weeks of age. Male mice were bred separately from their female counterparts and equal numbers of mice of both sexes were used in the experiments. Groups of matched pairs of cages were blindly allocated for experiments. A schematic illustration of the timeline of thiamine treatment is shown in S2 Fig. Homozygous KI mice were fed with a thiamine-restricted diet for 14 days, after which they were fed with a conventional diet. Homozygous KO mice were fed with a thiamine-restricted diet for 1, 2, or 3 days, after which they were fed with a conventional diet. In the experiment with high-thiamine–containing food, mice were first fed a thiamine-restricted diet for 2, 3, or 5 days, and then returned to a high-thiamine diet; recovered homozygous KO mice were fed with a high-thiamine diet and then reverted to a conventional diet at 60 days. The clinical signs used to monitor health and welfare included little body-weight gain and lethargy; these were monitored every day during the experimental period (60 days). Preliminary experiments reproducibly showed lethargy and weight loss, with death within 1–2 days, in model mice only. Lethargic mice did not recover after thiamine treatment. Epileptic seizures involving jerky movements of the limbs were not observed. Thus, in concordance with discussions with our attending veterinarian and the Animal Experimentation Committee of Aichi Human Service Centre, we considered lethargy as the end-point of the evaluation of thiamine treatment for SLC19A3 deficiency. After the experiment, mice were euthanized in accordance with the Guidelines for Animal Experimentation. Mice numbers (n) allocated for experiments are indicated. Research staff received training in animal care, handling, and welfare, organized by the animal experimentation committee of Aichi Human Service Centre.

### Determination of thiamine concentration in the blood and brain

Blood samples were collected by cardiac puncture and brain samples were prepared after perfusion with phosphate-buffered saline (PBS) under anesthesia with pentobarbital intraperitoneal injection (50 μg/g body weight). In cells, thiamine is converted to various thiamine derivatives, including TPP. Approximately 80–85% of thiamine in the brain is TPP [27]. Therefore, TPP is generally the more accurate indicator of thiamine deficiency. However, Slc19a3 transports thiamine from food into the bloodstream and then uptakes thiamine from blood into brain cells. To analyze the effect of thiamine transporter deficiency, measuring total thiamine—including the free form of thiamine, thiamine monophosphate, TPP, and thiamine triphosphate—is preferable to measuring TPP alone. For this reason, we determined total thiamine levels in the blood and brain. The concentrations of total thiamine in the blood and brain were determined by high-performance liquid chromatography (HPLC) post-label method using fluorescence detection according to Iwata *et al*. [28]. Briefly, 500 μL of 5% (w/v) trichloroacetic acid were added to 100 μL of blood and mixed vigorously. Brain was homogenized with four volumes of 50 mmol/L Tris-HCl (pH 7.5), and 80 μL of 32% (w/v) trichloroacetic acid were added to 320 μL of brain homogenate and mixed vigorously. The samples were centrifuged at 10,000 × *g* for 5 min, and subsequently, 40 μL of 1% (w/v) cyanogen bromide and 80 μL of 5% NaOH were added to 200 μL of the supernatant solution. After being kept for 10 min at room temperature, 50 μL of 1.5 mol/L HCl was added. The resulting samples were passed through a 0.45-μm microfilter equipped with Hydrophilic Durapore^TM^ (PVDF) (Millipore). The filtrate (50 μL) was directly injected into an HPLC system. A Tosoh ODS-100S (15 × 3.2 mm, I.D., average particle size: 5 μm) column was used as a pre-column, together with a Tosoh ODS-100S (250 × 4.6 mm, I.D., average particle size: 5 μm) column. Column temperature was maintained at 40°C. Samples were separated using the following mobile phase: 0.1 mol/L KH_2_PO_4_–K_2_HPO_4_ buffer (pH 7.0) containing 6% acetonitrile. Flow speed was set at 1.0 mL/min. Fluorescence intensities were measured with an excitation wavelength of 375 nm and emission wavelength of 430 nm with Shimadzu RF-10AXL [28].

### Detection of neurodegeneration, mature neurons, and astrocytes

The three *Slc19a3* KO and KI mice in each group were perfused with PBS under anesthesia with pentobarbital intraperitoneal injection (50 μg/g). Brains were fixed in 4% ice-cold paraformaldehyde/PBS perfusion and fixed at 4°C for 12 h. Next, the olfactory bulb and cerebellum were cut off and embedded in paraffin blocks. Serial 5-μm-thick coronal sections corresponding to images 67–69 in Allen Mouse Brain Atlas (http://www.brain-map.org/ [29]), and containing the submedial nucleus of the thalamus (SMT) region were cut and prepared for immunohistochemical staining using anti-NeuN (Chemicon #MAB377; Lot.2592741; 1:100) and anti-glial fibrillary acidic protein (GFAP) (DAKO #Z0334; Lot.096; 1:1000). Neurodegeneration was detected by Fluoro-Jade C–positive cells as described previously [30, 31]. Fluoro-Jade C staining was performed according to the manufacturer’s instructions (Biosensis). To standardize the anatomical structures found in all sections, grids were set on the SMT of the thalamus (0.13-mm^2^ circle), ventral anterior-lateral complex of the thalamus (VAL, 0.2-mm^2^ rectangle), and motor field of the cerebral cortex (0.2-mm^2^ rectangle) according to a brain map [29]. NeuN-immunoreactive cell nuclei in the grid were counted using Image J software.

### Statistical analysis

One-way analysis of variance (ANOVA), two-way ANOVA, and the Steel–Dwass test were used for statistical comparisons. The data are presented as the mean ± standard error of the mean (s.e.m.), and p < 0.05 denotes statistical significance.

## Results

### Effect of thiamine restriction on the survival of WT, KO and KI mice

**Fig 1:**
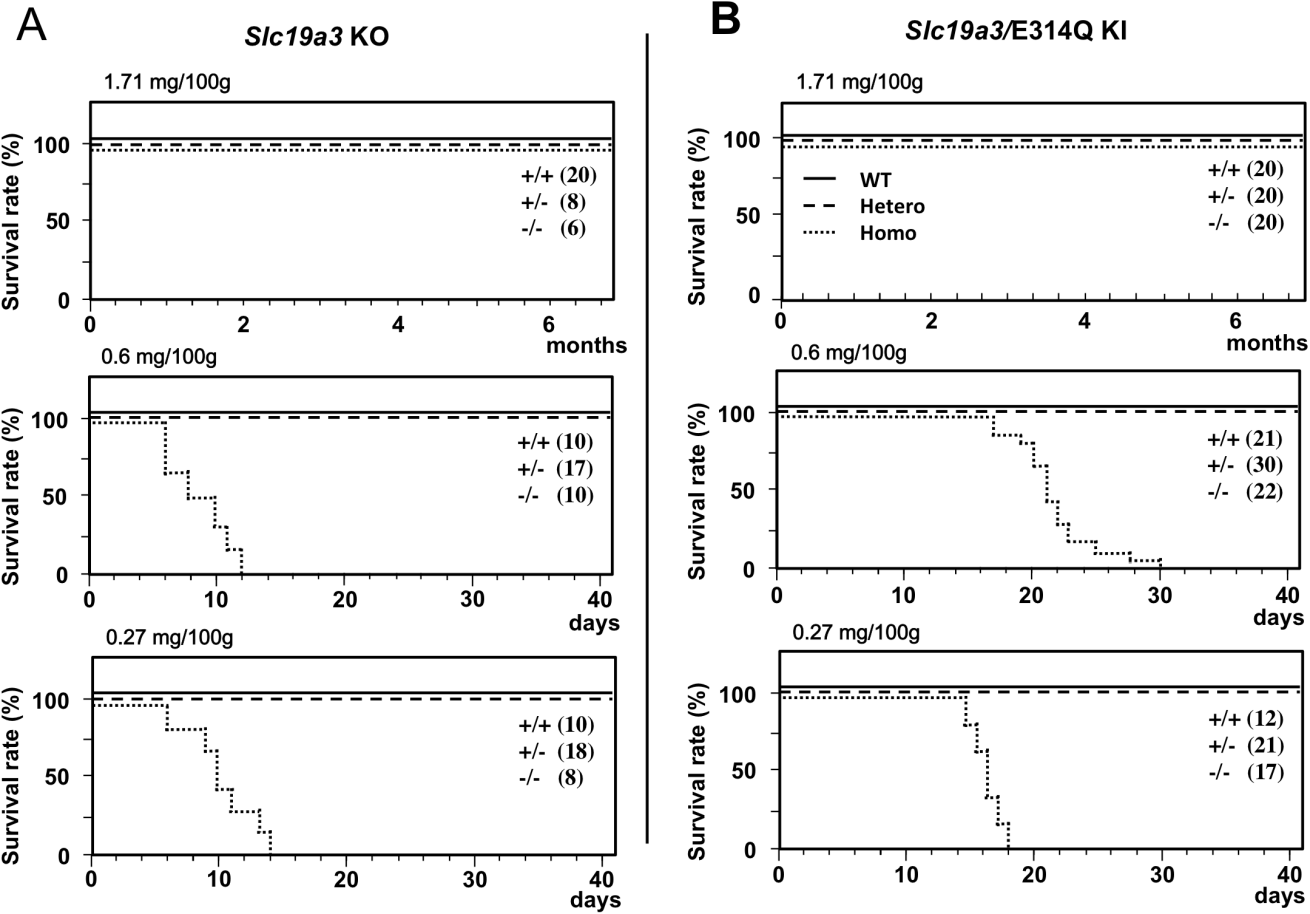
Survival rates of *Slc19a3* KO and KI mice fed with a conventional (thiamine: 1.71 mg/100 g food) and thiamine-deficient (thiamine: 0.60 or 0.27 mg/100 g food) diet. A. Survival rates of *Slc19a3* KO mice. B. Survival rates of *Slc19a3* KI mice. Thick lines, wild type (+/+); broken lines, heterozygous (+/-); and dotted lines, homozygous mice (-/-).

WT, homozygous, and heterozygous KO and KI mice fed a conventional diet (thiamine: 1.71 mg/100 g) survived for over 6 months without any phenotype of disease (Fig 1). Homozygous KO and KI mice fed a thiamine-restricted diet (thiamine: 0.60 mg/100 g food) showed paralysis, weight loss, and immobility, and died within 12 and 30 days, respectively. Similarly, homozygous KO and KI mice fed a thiamine-restricted diet with an even lower percentage of thiamine (thiamine: 0.27 mg/100g food) died within 14 and 18 days, respectively. However, WT and heterozygous KO and KI mice fed a thiamine-restricted diet (thiamine: 0.60 mg or 0.27 mg/100 g food) survived for over 6 months without any phenotype of disease (Fig 1).

### Effect of thiamine restriction on thiamine concentration in the blood and brain

**Fig 2:**
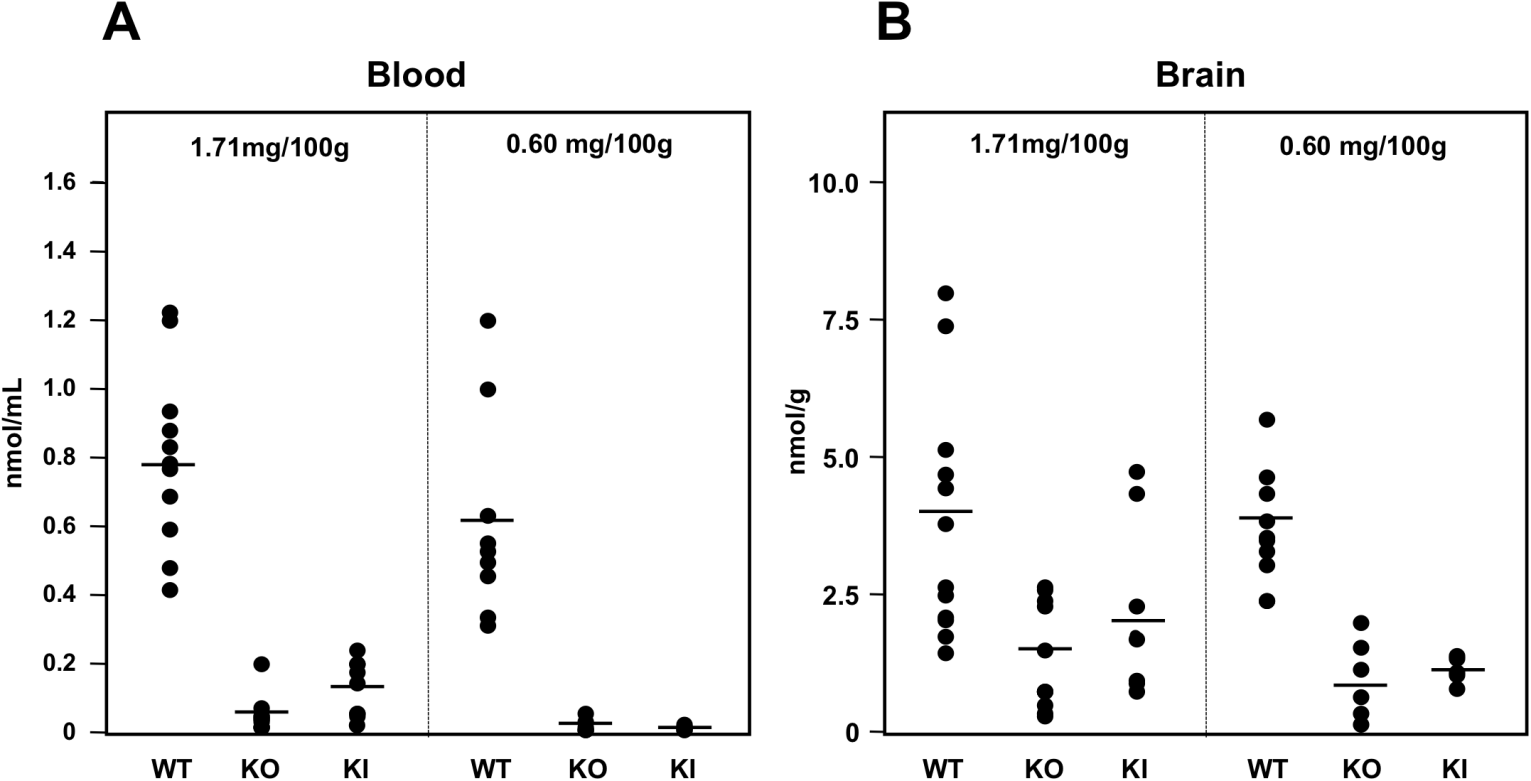
Thiamine concentrations in the blood and brain of WT, homozygous *Slc19a3* KO, and *Slc19a3* KI mice. Whole blood and cerebrum homogenates of KO and KI mice were obtained at 5 and 14 days of thiamine restriction, respectively. A. Thiamine concentration in whole blood (nmol/mL). B. Thiamine concentration in cerebrum homogenates (nmol/g wet weight). Bullets, individual thiamine concentrations; and bars, mean values.

Thiamine levels in the blood of homozygous KO and KI mice fed a conventional diet were decreased to 0.058 ± 0.051 and 0.126 ± 0.092 nmol/mL, respectively, at 7 weeks compared to WT mice (0.796 ± 0.259 nmol/mL) (Fig 2A). When WT and homozygous KO and KI mice were fed a thiamine-restricted diet (thiamine: 0.60 mg/100 g food), blood thiamine concentration at 5 and 14 days was markedly decreased to 0.010 ± 0.009 and 0.010 ± 0.006 nmol/mL, respectively, compared to WT mice (0.609 ± 0.288 nmol/mL) (Fig 2A). Thiamine concentration in brain homogenate of WT mice fed a conventional diet was 3.81 ± 2.18 nmol/g wet weight, and that of KO and KI was 1.33 ± 0.96 and 2.16 ± 1.55 nmol/g wet weight, respectively (Fig 2B). Notably, thiamine concentration in brain homogenate decreased steadily in KO and KI mice fed a thiamine-restricted diet (thiamine: 0.60 mg/100 g food) for 5 days (0.95 ± 0.72 nmol/g wet weight) and 14 days (1.11 ± 0.24 nmol/g wet weight), respectively, compared to WT (3.65 ± 1.02 nmol/g wet weight), before the mice presented any phenotype of disease (Fig 2B).

### NeuN-immunoreactive and Fluoro-Jade C–positive neurons in the SMT of thiamine-restricted homozygous KO and KI mice

**Fig 3:**
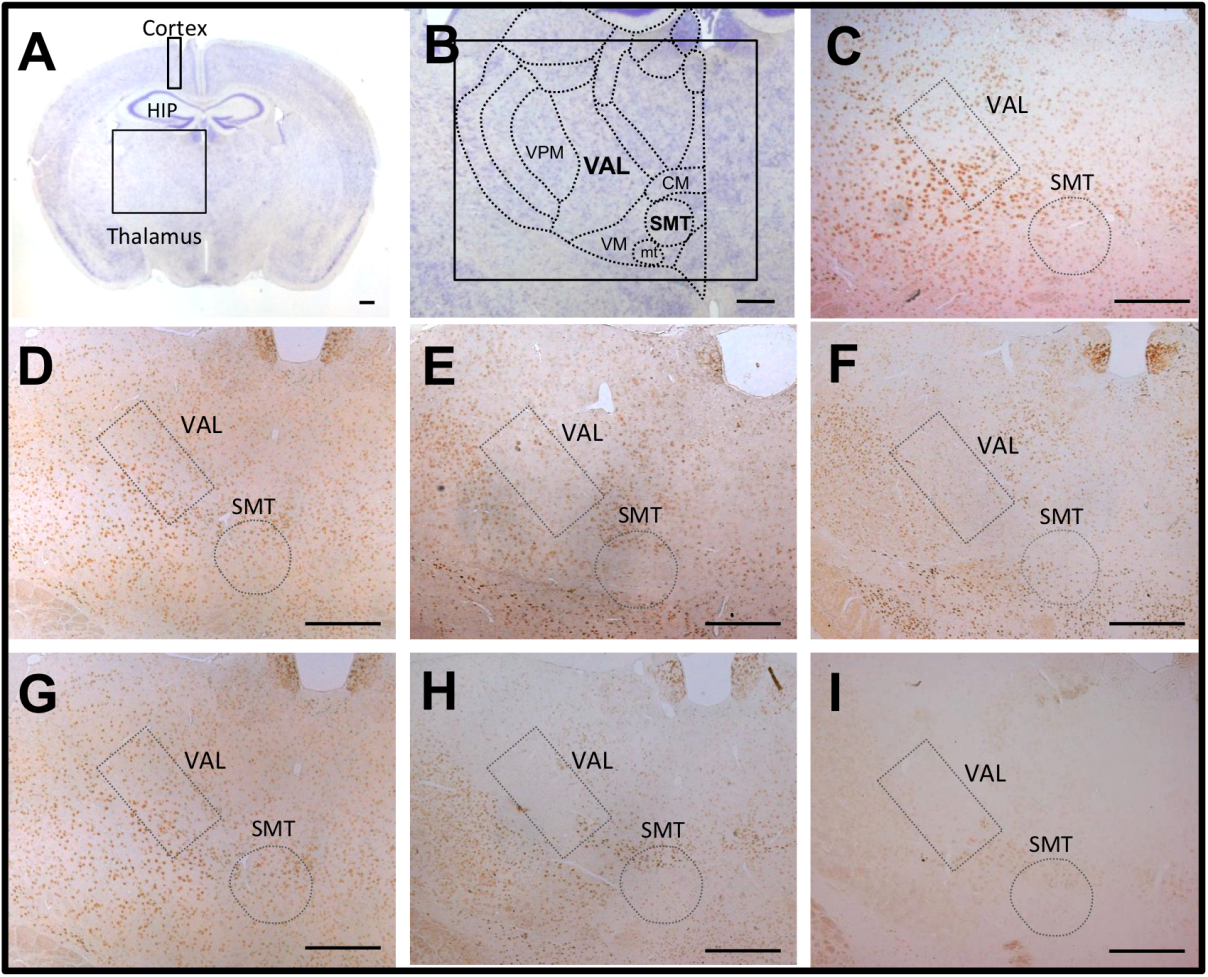
Temporal profile of NeuN-immunostaining in the hippocampus of WT, homozygous *Slc19a3* KO, and *Slc19a3* KI mice. NeuN-immunoreactive neurons in the submedial nucleus of the thalamus (SMT) and ventral anterior-lateral complex of the thalamus (VAL). A. Low magnification of a Nissl-stained brain slice at the level of the hippocampus (HIP). B. High-magnification overview of the specific regions of the thalamic area of a WT mouse. C. Closer view of a region contained in the inset of panel B. D–E. Brain slices of a homozygous KO mouse fed with thiamine 1.71 mg/100 g food for 12 days (D), thiamine 0.6 mg/100 g food for 5 days (E) and for 12 days (F). G–I. Brain slices of a homozygous KI mouse fed with thiamine 1.71 mg/100 g food for 14 days (G), thiamine 0.6 mg/100 g food for 14 days (H) and thiamine 0.27 mg/100 g food for 14 days (I). Scale bar, 200 μm. VPM: ventral posteromedial nucleus of the thalamus, CM: central medial nucleus of the thalamus, VM: ventral medial nucleus of the thalamus, mtt: mammillothalamic tract.

**Fig 4:**
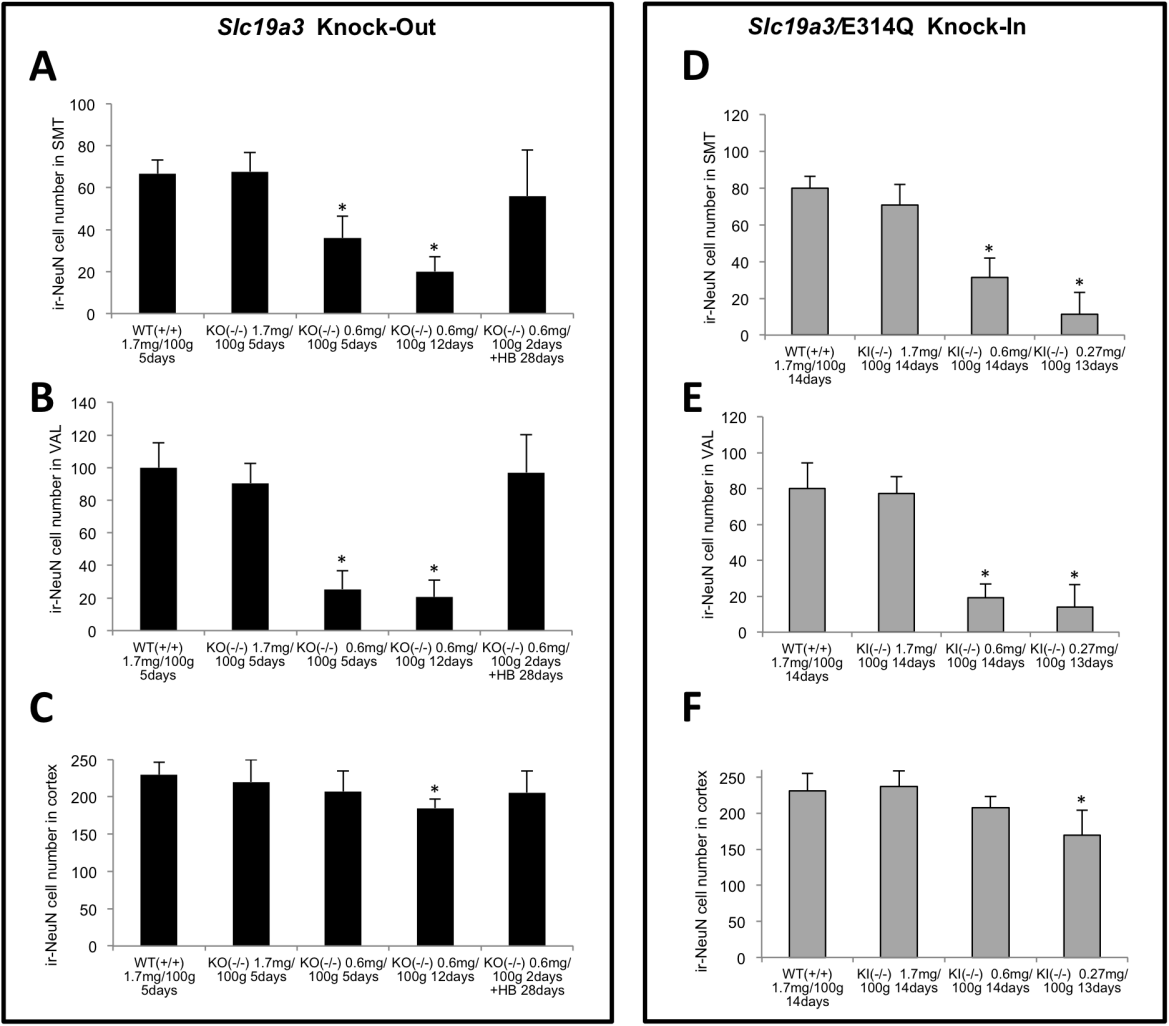
Numbers of NeuN-immunoreactive neurons in the thalamic area and cortex of WT, homozygous *Slc19a3* KO and KI mice. The numbers of NeuN-immunoreactive neurons of homozygous KO (A) and KI (D) mice in the submedial nucleus of the thalamus (SMT) (0.13 mm^2^); homozygous KO (B) and KI (E) mice in the ventral anterior-lateral complex of the thalamus (VAL) (0.2 mm^2^); and homozygous KO (C) and KI (F) mice in the cortex (0.2 mm^2^). Mean numbers of three experiments are shown. One-way ANOVA, two-way ANOVA, and the Steel–Dwass test were used for statistical comparisons. Asterisks (*) indicate that the numbers of NeuN-immunoreactive neurons of KO and KI mice fed with thiamine-restricted diets were significantly different from those of WT mice (first bar of each graph) at p < 0.05.

**Fig 5:**
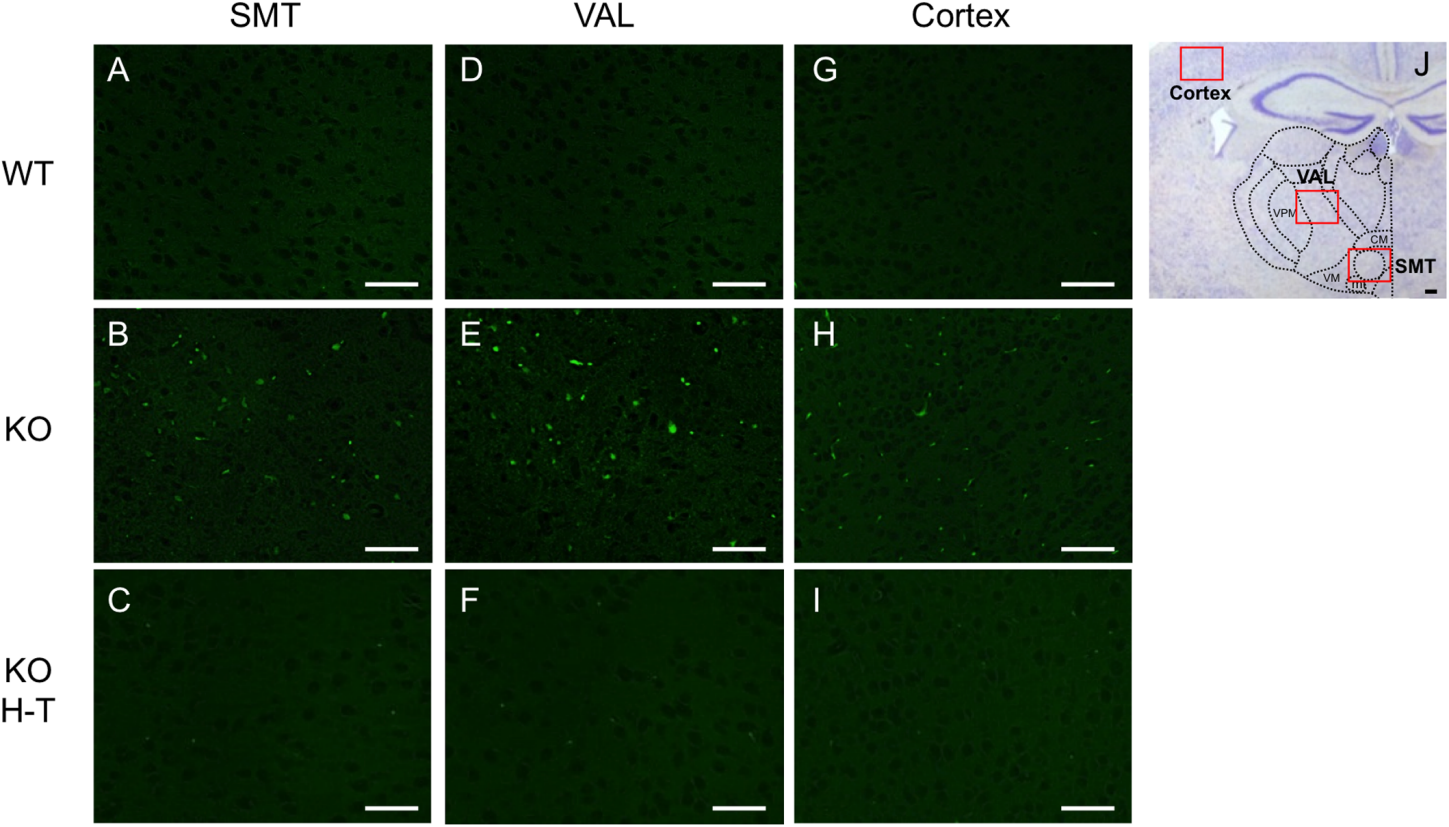
Fluoro-Jade C staining of WT, homozygous KO mice fed with a thiamine-restricted diet, and homozygous KO mice fed with a thiamine-restricted diet for 2 days and reverted to a high-thiamine diet. Brain slices of a WT and homozygous KO mouse fed with thiamine 1.71 mg/100 g food for 8 days, and surviving homozygous KO mice with high-thiamine diet showing the SMT (A, B, C), VAL (D, E, F), and cortex (G, H, I). Overview of the specific regions of the thalamic area of a WT mouse (J), with red boxes indicating the region of each part. Scale bar, 100 μm.

To analyze thiamine-restriction–induced brain lesions in homozygous KO and KI mice, brain slice specimens of each mouse were immunostained with an anti-NeuN antibody to detect mature neurons. NeuN-immunoreactive neurons were detected uniformly in the cortex and thalamic area of WT, heterozygous, and homozygous KO and KI mice fed a conventional diet (Fig 3C, 3D and 3G). After 5 days of a thiamine-restricted diet (thiamine: 0.60 mg/100 g food), the numbers of NeuN-immunoreactive neurons in the SMT and VAL of homozygous KO mice decreased to approximately 50% (Figs 3E, 4A and 4B). After 12 days of a thiamine-restricted diet, when most of homozygous KO mice had died of thiamine deficiency, the numbers of NeuN-immunoreactive neurons were remarkably decreased over a wide area of the thalamus, including the SMT (Figs 3F and 4A); in homozygous KI mice, the numbers of NeuN-immunoreactive neurons in the SMT and the dorsal area of the thalamus decreased to approximately 30% (Figs 3H and 4D). Similarly, NeuN-immunoreactive neurons were hardly detected in the thalamic area, including the SMT and VAL, in homozygous KI mouse fed a thiamine-restricted diet (thiamine: 0.27 mg/100 g food) for 14 days (Figs 3I, 4D and 4E). On the other hand, the decrease in NeuN-immunoreactive neurons in the cortex was less pronounced than in the SMT or VAL (Fig 4C and 4F). Next, to evaluate whether thiamine deficiency indeed caused neurodegeneration, we performed Fluoro-Jade C staining. We found that thiamine-restricted diet significantly increased the number of degenerating neurons in the SMT, VAL, and cortex compared to age-matched WT mice (Fig 5).

### GFAP expression in the thalamic area of KO and KI mice after thiamine restriction

**Fig 6:**
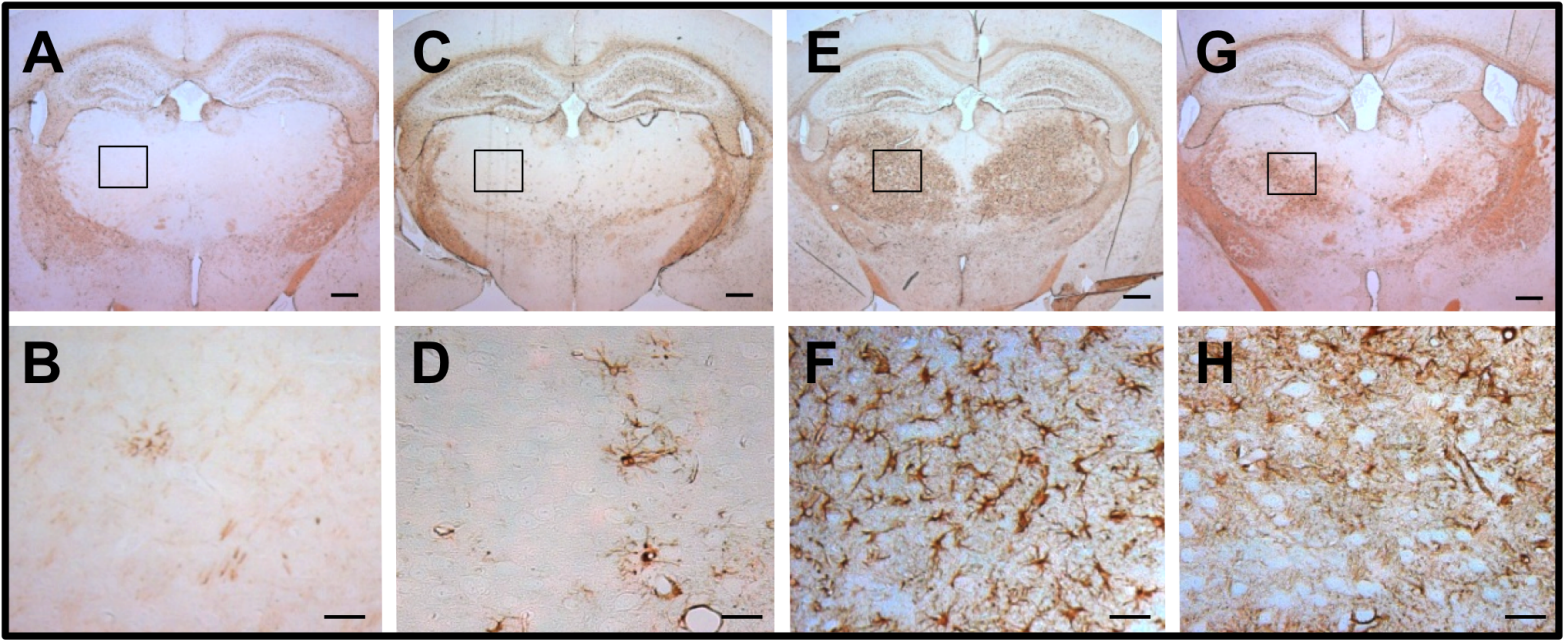
GFAP-immunoreactive astrocytes in the ventral anterior-lateral complex of the thalamus (VAL) of WT, homozygous *Slc19a3* KO, and *Slc19a3* KI mice. A, C, E, G. Low-magnification images of reactive astrocytes in the thalamus of a WT mouse fed with a conventional diet at postnatal day 35 (A); a homozygous KO mouse fed with thiamine 0.60 mg/100 g food for 5 days (C) and for 12 days (E); and a KI mouse fed with thiamine 0.60 mg/100 g for 14 days (G). B, D, F, and H are high-magnification images of the VAL area (insets) shown in panels of A, C, E, and G, respectively. Scale bars, 500 μm (upper panel) and 100 μm (lower panel).

Brains of homozygous KO and KI mice fed a thiamine-restricted diet were immunostained with an anti-GFAP antibody to analyze astrocyte activation. Immunoreactivity of GFAP remarkably increased in the bilateral thalamic area of homozygous KO and KI mice fed with a thiamine-restricted diet (thiamine: 0.6 mg/100 g food) for 12 or 14 days, compared to age-matched WT mice (Fig 6).

### Effect of thiamine restriction on high-thiamine–treated KO and KI recovered mice

**Fig 7:**
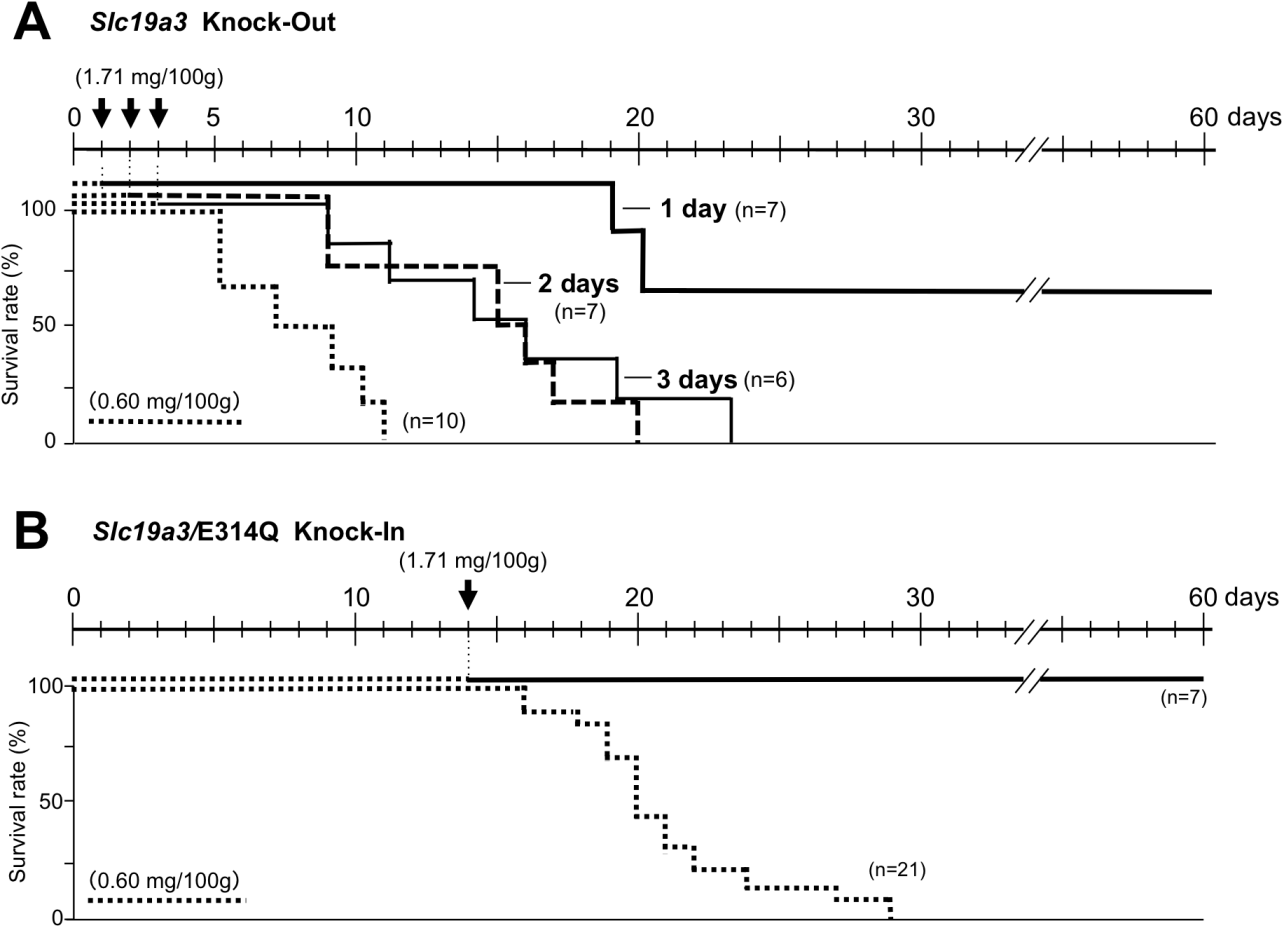
Survival rates of homozygous *Slc19a3* KO and KI mice fed with a conventional diet after thiamine restriction. A. Thirty homozygous KO mice were fed with a thiamine-restricted diet (thiamine 0.60 mg/100 g food) (dotted line). One (thick line), 2 (broken line), and 3 days (thin line) later, the diet of 7, 7, and 6 mice was reverted to a conventional diet, respectively. B. Twenty-eight homozygous KI mice were fed with thiamine 0.60 mg/100 g food (dotted line). After 14 days of a thiamine-restricted diet (thiamine 0.60 mg/100 g food), the diet of 7 homozygous KI mice was reverted to a conventional diet (1.71 mg/100 g food) (thick line).

**Fig 8:**
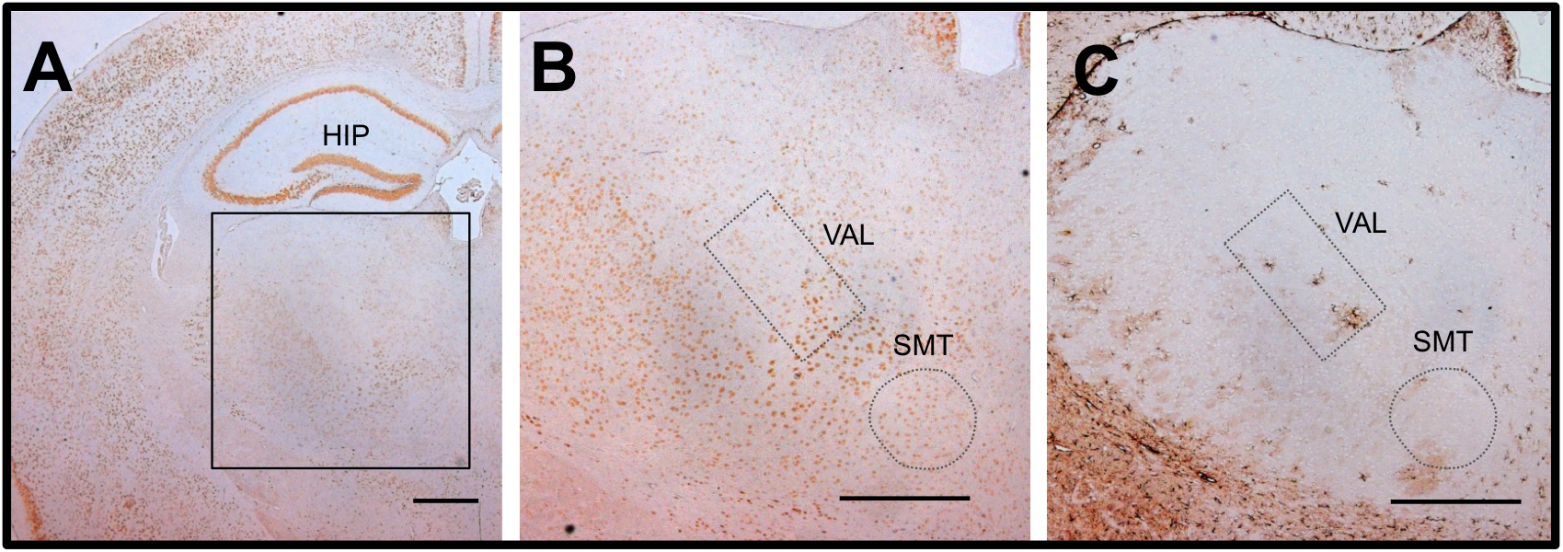
Recovery of homozygous *Slc19a3* KO mice with a high-thiamine diet after thiamine restriction. Homozygous KO mice were fed a thiamine-restricted diet (thiamine 0.60 mg/100 g food) for 2 days and then changed to a high-thiamine diet (thiamine 8.50 mg/100 g food) for 28 days. A. Low magnification of the NeuN-immunoreactive neurons in the thalamic area in a recovered homozygous KO mouse. B. High magnification of the inset of panel A. The submedial nucleus of the thalamus (SMT) and ventral anterior-lateral complex of the thalamus (VAL) are shown. C, GFAP immunostaining of the serial sections of B, respectively. Scale bar, 500 μm. The mean numbers of NeuN-immunoreactive neurons in the thalamic area and cortex of 3 homozygous KO mice are shown in Fig 4A–4C.

After 14 days of a thiamine-restricted food (thiamine: 0.60 mg/100 g food), all homozygous KI mice that were returned to a conventional diet (thiamine 1.71 mg/100 g) showed no phenotype of disease (Fig 7B). In contrast, when homozygous KO mice were fed a thiamine-restricted diet for 1, 2, and 3 days and then returned to a conventional diet, all mice died within the next 24 days, except the 1-day thiamine-restricted diet mice (Fig 7A). For 1-day thiamine-restricted food, some mice (4/7) recovered with conventional food (Fig 7A). To examine the effect of high-dose thiamine on the survival of homozygous KO mice, a high-thiamine diet (thiamine: 8.50 mg/100 g food) was prepared. After 2, 3, and 5 days of a thiamine-restricted diet (thiamine: 0.60 mg/100 g food), homozygous KO mice were fed a high-thiamine diet. The high-thiamine diet rescued most of the mice (10/12) and approximately half of the mice (7/13) that were fed thiamine-restriction food for 2 and 3 days, respectively. Notably, 3/7 mice survived after 5 days of a thiamine-restriction diet. Blood and brain thiamine concentrations of rescued mice, which recovered after 2 days of thiamine restriction, were 0.232 ± 0.077 nmol/mL and 1.12 ± 0.10 nmol/g at day 28 of the high-thiamine diet, respectively. Morphological findings of the brains of surviving homozygous KO mice on a high-thiamine diet after 2 days of thiamine restriction showed a slight decrease in NeuN-positive neurons compared to WT (Figs 8A, 8B and 4A–4C). The number of cells with significantly increased GFAP immunoreactivity was not increased in the vulnerable regions of the thalamus (Fig 8C). Fluoro-Jade C staining confirmed that a high-thiamine diet prevented acute neurodegeneration (Fig 5C, 5F and 5I). The mean thiamine concentrations in the blood and brain of five homozygous KO mice and three WT mice fed a high-thiamine diet for 28 days after 2 days of thiamine restriction were 0.232 nmol/mL and 1.12 nmol/g wet weight, and 0.540 nmol/mL and 2.32 nmol/g wet weight, respectively.

## Discussion

To evaluate the therapeutic effects and problems associated with high-thiamine treatment in THMD2, and to evaluate its pathogenesis, we performed biochemical and immunohistochemical studies of *Slc19a3* KI mice and previously reported *Slc19a3* KO mice fed a conventional, thiamine-restriction diet, or high-thiamine diet, and compared their phenotypes to those of previously reported TD mice. In the present study, we firstly demonstrated that homozygous *Slc19a3* KO and KI mice fed a thiamine-restricted diet died within several weeks with specific brain lesions. In contrast, WT and heterozygous KO and KI mice fed the same diet showed no abnormalities. Thus, homozygous KO and KI mice are useful models of *SLC19A3* deficiency (THMD2).

Homozygous KO mice first generated by Reidling *et al*. showed no phenotype of disease when fed with a conventional diet until 10 months of age [24]. The authors concluded that Slc19a3 is required for normal uptake of thiamine in the intestine from the following findings: 1) the blood concentration of thiamine in homozygous KO mice decreased to approximately 70% that of WT mice, and 2) thiamine uptake by intestinal epithelial cells and intestinal loops in the KO mice decreased to 20–30% that of WT mice, but biotin uptake by intestinal loops in the KO mice was not changed [24]. These results are consistent with a previous report that SLC19A3 is expressed predominantly at upper intestine [6]. In our study, thiamine concentrations in the blood of homozygous *Slc19a3* KO and KI mice fed a conventional diet (thiamine 1.71 mg/100 g) were dramatically decreased to 7% and 15%, respectively, compared to that in WT mice (Fig 2). These findings indicate that E320Q mutation is a pathological mutation and SLC19A3 E320Q has only a small amount of thiamine transporter activity in the intestine. However, residual transporter activity prolonged the survival of homozygous KI mice compared to homozygous KO mice when fed a thiamine-restricted diet (Fig 1).

The blood thiamine concentration is mainly regulated by the balance of upper intestine absorption and urinary excretion and reabsorption, and approximately 53% of thiamine is excreted through the urine [32, 33]. Urinary thiamine excretion is regulated by MATE1 and MATE2-K [34] and reabsorption is performed by SLC19A2 and SLC19A3 in renal epithelial cells [35]. Thus, decreased steady-state level of blood thiamine concentration of homozygous KO and KI mice fed with a conventional diet is strongly associated with the null or defective Slc19a3 at the upper intestine and renal epithelial cells. When homozygous KO or KI mice eat 4 g of conventional food and drink 6 mL of water per day [36], the mean concentration of thiamine is 11.4 μg/mL (33.8 μM) in the intestine. Since thiamine is absorbed by passive diffusion at high intraluminal concentrations (>2 μM) [37, 38], the small amount of blood thiamine (7% that of the WT) of homozygous KO mice fed a conventional diet could be from the thiamine absorption by other transporters including Slc19a2 (Km of 2.5 μM) and passive diffusion. Differences in blood thiamine concentration in KO mice between a previous report [24] and this study using the same KO mice are, therefore, likely due to the thiamine content of food (previously reported mice may have been fed more thiamine-containing food than in this study). When homozygous KO and KI mice were fed a thiamine-restricted diet (thiamine: 0.60 mg/100 g food), thiamine blood concentrations decreased to approximately 1.6% that of WT, which were lethal for both mice. Therefore, there could be a minimum blood thiamine concentration (threshold) necessary for survival of mice harboring mutant Slc19a3. The presented study demonstrated that the threshold of blood thiamine concentration is between approximately 0.01 and 0.06 nmol/mL (the former is fatal for homozygous *Slc19a3* KO mice and the KO mice survive with the latter) (Fig 2).

The thiamine content of brain is critical for the survival of mice. TD mice show symptoms of disease when cerebral cortex and thalamus thiamine concentration is decreased to approximately 15% that of control mice [39]. In this study, the thiamine content of the brain was relatively conserved compared to that of blood in a thiamine-restricted diet (Fig 2). Approximately 80–85% of thiamine in the brain is TPP [27], and most of the TPP binds to apoenzymes and remains in the neuronal cells [40]. Therefore, thiamine content in the brain could slowly decrease in homozygous KO and KI mice when they started a thiamine-restricted diet. The percentage of brain thiamine content of the homozygous KO and KI mice vs. WT mice was 27% and 29% on days 5 and 14, respectively. It is possible that the percentage of thiamine in the brain thiamine could be less than 15% at the time of death.

The most important pathological features of TD mice are the disappearance of NeuN-positive neurons in the SMT and VAL after 11 days of TD treatment (TD11) [15]. Swelling of astrocytes results in the release of glutamate, increased extracellular levels of glutamate, and downregulation of astrocytic glutamate transporters GLT-1 and GLAST at the vulnerable regions of the thalamus [41–44]. The decreased levels of GLT-1 and GLAST were also reported in brains with Wernicke encephalopathy caused by thiamine deficiency [45]. These morphological and biochemical changes of astrocytes caused by injury or disease are referred to as astrogliosis. The hallmarks of astrogliosis are hypertrophy and upregulation of the intermediate filament GFAP. This is because a subset of reactive astrocytes re-enter the cell cycle and appear to revert to an immature phenotype [46]. The activation of astrocytes and microglial cells in the vulnerable regions of the thalamus has been reported in early stages of TD mice [47]. In this study, we showed that severe neurodegeneration in the SMT and VAL occurred in both homozygous KO and KI mice fed a thiamine-restricted diet (Figs 3E, 3F, 3H, 3I and 4). Astrogliosis and microglial activation have also observed at the vulnerable regions of homozygous KO mice (Fig 6, S3 Fig). These findings are consistent with autopsy brain findings in which significant neuronal loss and astrogliosis have been observed in two patients with THMD2 [20]. Taken together, the hallmarks of brain pathological findings of THMD2 and model mice are neurodegeneration, and astrogliosis caused primary by TPP deficiency and decreased activities of TPP-dependent enzymes (e.g., αKGDH in the TCA cycle) [48].

We found that high-thiamine–containing food has great impact on the recovery of mice fed a thiamine-restricted diet for a few days. Pathological studies showed that administration of high thiamine prevented neurodegeneration (Fig 5C, 5F and 5I) and astrogliosis associated with activated astrocytes (Fig 8). This result suggests that the affected neurons of patients with THMD2 have potential to recover, and that the high amount of TPP in the brain is essential for treatment. Therefore, patients with *SLC19A3* null mutations need a large amount of thiamine in the brain, where TPP generated from thiamine is absorbed by other factors, including SLC19A2 and passive diffusion. We also found that the recovered homozygous KO mice fed high thiamine food showed exacerbated phenotypes (i.e., the recovered mice showed similar rates of mortality as those noted in mice fed with a thiamine restricted diet), when the amount of thiamine was decreased to the amount in conventional food. This result proposes the important question that how long the patients with THMD2 should continue high dose thiamine treatment by monitoring blood thiamine concentration, or how to decrease the thiamine concentration after the recovery of the symptoms.

Mean brain thiamine concentration of 5 homozygous KO mice fed with high-thiamine food for 28 days after 2 days of thiamine restriction is 1.12 nmol/g wet weight. This concentration is approximately 48% (>15%) of that of three WT mice (2.32 nmol/g wet weight) fed a high-thiamine diet. Brain thiamine concentration of WT mice fed a conventional and high-thiamine diet was similar, suggesting that those amounts were the upper limit of thiamine concentration in the brain. The increased thiamine (TPP) concentration of homozygous KO brains of mice fed with a high-thiamine diet could be due to the increased thiamine concentration in the blood (0.232 nmol/mL = 0.23 μM). In the brain of these mice, thiamine is possibly transported by Slc19a2 to the neurons, because passive transport of thiamine in the brain may occur when thiamine concentration is greater than 2 μM [35]. However, there is a possibility that passive diffusion may be affected by blood–brain barrier abnormalities [49, 50]. Thus, Slc19a2 may play an important role in the transport of thiamine in the brain of homozygous KO mice.

The newly identified exacerbated phenotype of recovered homozygous KO mice with high-thiamine diet indicates that the brain is damaged within only 2 days of thiamine restriction in KO mice and that sufficient thiamine is not incorporated into the brain after the mice are reverted to a conventional diet. Previous studies demonstrated that 1) SLC19A3 is expressed within cerebral blood vessels at the basement membrane and in perivascular pericytes [20]; 2) blood–brain barrier abnormalities is a characteristic feature of TD mice and rats [49, 50]; and 3) intercellular adhesion molecule 1 (ICAM-1) induction in endothelial cells of microvessels of the thalamus is the early trigger of TD-induced neurodegeneration in TD mice [51]. Cerebrovascular disturbance of these mechanisms may occur in KO mice with a thiamine-restricted diet. Therefore, cerebrovascular damage in KO mice is likely one of the causes of reduced thiamine uptake into neuronal cells when a high-thiamine diet is changed to a conventional diet. Significant increases of activated microglial cells have also reported in medial thalamic nuclei of TD rats prior to any evidence of increased immunoglobulin G immunostaining or significant neuronal cell loss [52]. CD40-positive microglia and CD40–CD40L interactions promote neurodegeneration in early stages of TD in mice [47]. We detected significant increases of activated microglial cells in homozygous KO mice fed with a thiamine-restricted diet for 8 days in whom neurodegeneration had occurred (S3 Fig). Thus, the increases of activated microglial cells are also suggestive of an exacerbated phenotype of recovered homozygous KO mice with high-thiamine diet. Further studies are necessary to clarify the molecular mechanism of early neuronal damages caused by 2 days of thiamine restriction, for example, by analyzing the proteins (e.g., Slc19a2, ICAM-1, and CD40) expressed in the brain of *Slc19a3*-deficient mice.

The results of this study are very important findings concerning the treatment of THMD2. We suggest that when patients with THMD2 recover after administration of high-dose thiamine, doctors should carefully reduce the amount of thiamine by monitoring the blood concentration of thiamine.

## Supporting information

S1 Fig Generation of Slc19a3 E314Q KI mice. A. Schematic illustration of the construction strategy of Slc19a3 E314Q KI mice. The targeting vector contains a 3.0-kb 5' arm containing exon 3 with a neomycin selection cassette flanked by loxP sites and a 8.1-kb 3' arm containing exon 4–5 with the E314Q knock-in mutation (c.940G>C, [p.E314Q]) in exon 4. B. Confirmation of the genome sequence of homozygous E314Q KI mice. The mutation (c.940G>C, [p.E314Q]) in exon 4 was validated. The c.940G>C substitution generated a novel *Rsa*I site (GTAC, underlined). C. Genotyping by PCR-RFLP analysis using *Rsa*I. A primer pair (sense S1: 5′-agcaacccagatccagaaaat-3′; antisense A1: 5′-acacttacctccaaatgttgc-3′) was used to amplify a part of exon 4. *Rsa*I was used to digest the 240-bp mutant (c.940G>C) PCR product, which generated the 209- and 31-bp PCR products. Lane 1, WT; lane 2, heterozygous E314Q KI mouse; lane 3, homozygous E314Q KI mouse.
(TIF)

S2 Fig Schematic illustration of the timeline of thiamine treatment. KI experiment 1: Homozygous KI mice aged 5–6 weeks were fed with a thiamine-restricted diet for 14 days and then changed to a conventional diet. KO experiments 1–3: Homozygous KO mice aged 5–6 weeks were fed with a thiamine-restricted diet for 1, 2, or 3 days and then changed to a conventional diet. KO experiments 4–6: Homozygous KO mice aged 5–6 weeks were fed with a thiamine-restricted diet for 2, 3, or 5 days and then changed to a high-thiamine diet. In KO experiment 4, recovered homozygous KO mice were reverted to a conventional diet at 60 days.
(TIF)

S3 Fig Iba1-immunopositive microglial cells in the VAL of WT (B) and homozygous KO mice (C) fed with thiamine-restricted diet for 8 days. Overview of the specific regions of the thalamic area of a WT mouse (A), with the red box indicating the region of VAL. Anti-Iba1 antibody (Wako Pure Chemical Industries, #019–19741, 1:500) was used. Scale bar, 200 μm. Note that homozygous KO mice (C) exhibit an increase in the number of activated microglia, which have larger cell bodies and thicker processes.
(TIF)

## References

[1] McCormick DB. Thiamine In: Shils ME, Young VR, editors. Modern nutrition in health and disease. Philadelphia: Lea and Febiger; 1988 pp. 355–361.

[2] Anderson SH, Vickery CA, Nicol AD. Adult thiamine requirements and the continuing need to fortify processed cereals. Lancet 1986; 2: 85–89. 2873388 10.1016/s0140-6736(86)91618-1

[3] Diaz GA, Banikazemi M, Oishi K, Desnick RJ, Gelb BD. Mutations in a new gene encoding a thiamine transporter cause thiamine-responsive megaloblastic anaemia syndrome. Nat Genet 1999; 22: 309–312. doi: 10.1038/10385 10391223 10.1038/10385

[4] Eudy JD, Spiegelstein O, Barber RC, Wlodarczyk BJ, Talbot J, Finnell RH. Identification and characterization of the human and mouse SLC19A3 gene: a novel member of the reduced folate family of micronutrient transporter genes. Mol Genet Metab 2000; 71: 581–590. doi: 10.1006/mgme.2000.3112 11136550 10.1006/mgme.2000.3112

[5] Zhao R, Goldman ID. Folate and thiamine transporters mediated by facilitative carriers (SLC19A1-3 and SLC46A1) and folate receptors. Mol Aspects Med 2013; 34: 373–385. doi: 10.1016/j.mam.2012.07.006 23506878 10.1016/j.mam.2012.07.006 PMC3831518

[6] Said HM, Balamurugan K, Subramanian VS, Marchant JS. Expression and functional contribution of hTHTR-2 in thiamin absorption in human intestine. Am J Physiol 2004; 286: 491–498. 10.1152/ajpgi.00361.2003 14615284

[7] Nosaka K, Onozuka M, Kakazu N, Hibi S, Nishimura H, Nishino H, et al Isolation and characterization of a human thiamine pyrophosphokinase cDNA. Biochem Biophys Acta 2001; 1517: 293–297. 11342111 10.1016/s0167-4781(00)00247-5

[8] Zhao R, Gao F, Goldman ID. Molecular cloning of human thiamin pyrophosphokinase. Biochim Biophys Acta 2001; 1517: 320–322. 11342117 10.1016/s0167-4781(00)00264-5

[9] Berdanier CD. Water-soluble vitamins In: Berdanier CD, editor. Advanced Nutrition-Micronutrients. New York: CRC Press; 1998 pp. 73–124.

[10] Woolley DW. An enzymatic study of the mode of action of pyrithiamine (neopyrithiamine). J Biol Chem 1951; 191: 43–54. 14850443

[11] Rindi G, Perri V. Uptake of pyrithiamine by tissue of rats. Biochem J 1961; 80: 214–216. 13741739 10.1042/bj0800214 PMC1243973

[12] Watanabe I. Pyrithiamine-induced acute thiamine-deficient encephalopathy in the mouse. Exp Mol Pathol 1978; 28: 381–394. 648625 10.1016/0014-4800(78)90012-6

[13] Zhang SX, Weilersbacher GS, Henderson SW, Corso T, Olney JW, Langlais PJ. Excitotoxic cytopathology, progression, and reversibility of thiamine deficiency-induced diencephalic lesions. J Neuropathol Exp Neurol 1995; 54: 255–267. 7876893 10.1097/00005072-199503000-00012

[14] Hazell AS, Todd KG, Butterworth RF. Mechanisms of neuronal cell death in Wernicke’s encephalopathy. Metab Brain Dis 1998; 13: 97–122. 9699919 10.1023/a:1020657129593

[15] Ke ZJ, DeGiorgio LA, Volpe BT, Gibson GE. Reversal of thiamine deficiency-induced neurodegeneration. J Neuropathol Exp Neurol 2003; 62: 195–207. 12578229 10.1093/jnen/62.2.195

[16] Abdou E, Hazell AS. Thiamine deficiency: an update of pathophysiologic mechanisms and future therapeutic considerations. Neurochem Res 2015; 40: 353–361. doi: 10.1007/s11064-014-1430-z 25297573 10.1007/s11064-014-1430-z

[17] Ozand PT, Gascon GG, Al Essa M, Joshi S, Al Jishi E, Bakheet S, et al Biotin-responsive basal ganglia disease: a novel entity. Brain 1998; 121: 1267–1279. 9679779 10.1093/brain/121.7.1267

[18] Zeng WQ, Al-Yamani E, Acierno JS Jr, Slaugenhaupt S, Gillis T, MacDonald ME, et al Biotin-responsive basal ganglia disease maps to 2q36.3 and is due to mutations in SLC19A3. Am J Hum Genet 2005; 77: 16–26. doi: 10.1086/431216 15871139 10.1086/431216 PMC1226189

[19] Yamada K, Miura K, Hara K, Suzuki M, Nakanishi K, Kumagai T, et al A wide spectrum of clinical and brain MRI findings in patients with *SLC19A3* mutations. BMC Med Genet 2010; 11: 171 doi: 10.1186/1471-2350-11-171 21176162 10.1186/1471-2350-11-171 PMC3022826

[20] Kevelam SH, Bugiani M, Salomons GS, Feigenbaum A, Blaser S, Prasad C, et al Exome sequencing reveals mutated *SLC19A3* in patients with an early-infantile, lethal encephalopathy. Brain 2013; 136: 1534–1543. doi: 10.1093/brain/awt054 23482991 10.1093/brain/awt054

[21] Gerards M, Kamps R, van Oevelen J, Boesten I, Jongen E, de Koning B, et al Exome sequencing reveals a novel Moroccan founder mutation in *SLC19A3* as a new cause of early-childhood fatal Leigh syndrome. Brain 2013; 136: 882–890. doi: 10.1093/brain/awt013 23423671 10.1093/brain/awt013

[22] Debs R, Depienne C, Rastetter A, Bellanger A, Degos B, Galanaud D, et al Biotin-responsive basal ganglia disease in ethnic Europeans with novel *SLC19A3* mutations. Arch Neurol 2010; 67: 126–130. doi: 10.1001/archneurol.2009.293 20065143 10.1001/archneurol.2009.293

[23] Kono S, Miyajima H, Yoshida K, Togawa A, Shirakawa K, Suzuki H. Mutations in a thiamine-transporter gene and Wernicke's-like encephalopathy. N Engl J Med 2009; 360: 1792–1794. doi: 10.1056/NEJMc0809100 19387023 10.1056/NEJMc0809100

[24] Reidling JC, Lambrecht N, Kassir M, Said HM. Impaired intestinal vitamin B1 (thiamin) uptake in thiamin transporter-2-deficient mice. Gastroenterology 2010; 138: 1802–1809. doi: 10.1053/j.gastro.2009.10.042 19879271 10.1053/j.gastro.2009.10.042 PMC4916904

[25] Tabarki B, Alfadhel M, AlShahwan S, Hundallah K, AlShafi S, AlHashem A. Treatment of biotin-responsive basal ganglia disease: Open comparative study between the combination of biotin plus thiamine versus thiamine alone. Eur J Paediatr Neurol 2015; 19: 547–552. doi: 10.1016/j.ejpn.2015.05.008 26095097 10.1016/j.ejpn.2015.05.008

[26] Reeves PG, Nielsen FH, Fahey GC Jr. AIN-93 purified diets for laboratory rodents: final report of the American Institute of Nutrition ad hoc writing committee on the reformulation of the AIN-76A rodent diet. J Nutr 1993; 123: 1939–1951. 8229312 10.1093/jn/123.11.1939

[27] Bettendorff L, Peeters M, Wins P, Schoffeniels E. Metabolism of thiamine triphosphate in rat brain: correlation with chloride permeability. J Neurochem 1993; 60: 423–434. 8380431 10.1111/j.1471-4159.1993.tb03168.x

[28] Iwata H, Matsuda T, Tonomura H. Improved high-performance liquid chromatographic determination of thiamine and its phosphate esters in animal tissues. J Chromatogr 1988; 450: 317–323. 3241017 10.1016/s0021-9673(01)83586-x

[29] Allen PG. (2006). ALLEN BRAIN ATLAS. Available from: http://www.brain-map.org/.

[30] Schmuck G, Kahl R. The use of Fluoro-Jade in primary neuronal cell cultures. Arch Toxicol 2009; 83: 397–403. doi: 10.1007/s00204-008-0360-4 18815771 10.1007/s00204-008-0360-4

[31] Noraberg J, Kristensen BW, Zimmer J. Markers for neuronal degeneration in organotypic slice cultures. Brain Res Brain Res Protoc. 1999; 3: 278–290. 9974143 10.1016/s1385-299x(98)00050-6

[32] Tallaksen CM, Bell H, Bøhmer T. Thiamin and thiamin phosphate ester deficiency assessed by high performance liquid chromatography in four clinical cases of Wernicke encephalopathy. Alcohol Clin Exp Res 1993; 17: 712–716. 8333605 10.1111/j.1530-0277.1993.tb00825.x

[33] Losa R, Sierra MI, Fernández A, Blanco D, Buesa JM. Determination of thiamine and its phosphorylated forms in human plasma, erythrocytes and urine by HPLC and fluorescence detection: a preliminary study on cancer patients. J Pharm Biomed Anal 2005; 37: 1025–1029. doi: 10.1016/j.jpba.2004.08.038 15862682 10.1016/j.jpba.2004.08.038

[34] Kato K, Mori H, Kito T, Yokochi M, Ito S, Inoue K, et al Investigation of endogenous compounds for assessing the drug interactions in the urinary excretion involving multidrug and toxin extrusion proteins. Pharm Res 2014; 31: 136–147. doi: 10.1007/s11095-013-1144-y 23907530 10.1007/s11095-013-1144-y

[35] Ashokkumar B, Vaziri ND, Said HM. Thiamin uptake by the human-derived renal epithelial (HEK-293) cells: cellular and molecular mechanisms. Am J Physiol Renal Physiol 2006; 291: 796–805. 10.1152/ajprenal.00078.2006 16705148

[36] Bachmanov AA, Reed DR, Beauchamp GK, Tordoff MG. Food intake, water intake, and drinking spout side preference of 28 mouse strains. Behav Genet 2002; 32: 435–443. 12467341 10.1023/a:1020884312053 PMC1397713

[37] Hayashi K, Yoshida S, Kawasaki T. Thiamine transport in the brush border membrane vesicles of the guinea-pig jejunum. Biochim Biophys Acta 1981; 641: 106–113. 6260179 10.1016/0005-2736(81)90573-3

[38] Said HM, Ortiz A, Kumar CK, Chatterjee N, Dudeja PK, Rubin S. Transport of thiamine in human intestine: mechanism and regulation in intestinal epithelial cell model Caco-2. Am J Physiol 1999; 277: 645–651. 10.1152/ajpcell.1999.277.4.C645 10516094

[39] Héroux M, Butterworth RF. Regional alterations of thiamine phosphate esters and of thiamine diphosphate-dependent enzymes in relation to function in experimental Wernicke's encephalopathy. Neurochem Res 1995; 20: 87–93. 7739764 10.1007/BF00995157

[40] Bettendorff L. The compartmentation of phosphorylated thiamine derivatives in cultured neuroblastoma cells. Biochim Biophys Acta 1994; 1222: 7–14. 8186267 10.1016/0167-4889(94)90019-1

[41] Hazell AS, Butterworth RF, Hakim AM. Cerebral vulnerability is associated with selective increase in extracellular glutamate concentration in experimental thiamine deficiency. J Neurochem 1993; 61: 1155–1158. 8103080 10.1111/j.1471-4159.1993.tb03635.x

[42] Kimelberg HK, Rutledge E, Goderie S, Charniga C. Astrocytic swelling due to hypotonic or high K^+^ medium causes inhibition of glutamate and aspartate uptake and increases their release. J Cereb Blood Flow Metab 1995; 15: 409–416. doi: 10.1038/jcbfm.1995.51 7713998 10.1038/jcbfm.1995.51

[43] Langlais PJ, Zhang SX. Extracellular glutamate is increased in thalamus during thiamine deficiency-induced lesions and is blocked by MK-801. J Neurochem 1993; 61: 2175–2182. 8245970 10.1111/j.1471-4159.1993.tb07457.x

[44] Hazell AS, Rao KV, Danbolt NC, Pow DV, Butterworth RF. Selective down-regulation of the astrocyte glutamate transporters GLT-1 and GLAST within the medial thalamus in experimental Wernicke's encephalopathy. J Neurochem 2001; 78: 560–568. 11483659 10.1046/j.1471-4159.2001.00436.x

[45] Hazell AS, Sheedy D, Oanea R, Aghourian M, Sun S, Jung JY, et al Loss of astrocytic glutamate transporters in Wernicke encephalopathy. Glia 2010; 58: 148–156. doi: 10.1002/glia.20908 19565658 10.1002/glia.20908 PMC3388119

[46] Robel S, Sontheimer H. Glia as drivers of abnormal neuronal activity. Nat Neurosci 2016; 19: 28–33. doi: 10.1038/nn.4184 26713746 10.1038/nn.4184 PMC4966160

[47] Ke ZJ, Calingasan NY, DeGiorgio LA, Volpe BT, Gibson GE. CD40-CD40L interactions promote neuronal death in a model of neurodegeneration due to mild impairment of oxidative metabolism. Neurochem Int 2005; 47: 204–215. doi: 10.1016/j.neuint.2005.03.002 15885854 10.1016/j.neuint.2005.03.002

[48] Gibson GE, Ksiezak-Reding H, Sheu KF, Mykytyn V, Blass JP. Correlation of enzymatic, metabolic, and behavioral deficits in thiamin deficiency and its reversal. Neurochem Res 1984; 9: 803–814. 6149477 10.1007/BF00965667

[49] Harata N, Iwasaki Y. Evidence for early blood-brain barrier breakdown in experimental thiamine deficiency in the mouse. Metab Brain Dis 1995; 10: 159–174. 7675014 10.1007/BF01991863

[50] Calingasan NY, Baker H, Sheu KF, Gibson GE. Blood-brain barrier abnormalities in vulnerable brain regions during thiamine deficiency. Exp Neurol 1995; 134: 64–72. doi: 10.1006/exnr.1995.1037 7672039 10.1006/exnr.1995.1037

[51] Calingasan NY, Huang PL, Chun HS, Fabian A, Gibson GE. Vascular factors are critical in selective neuronal loss in an animal model of impaired oxidative metabolism. J Neuropathol Exp Neurol 2000; 59: 207–217. 10744059 10.1093/jnen/59.3.207

[52] Todd KG, Butterworth RF. Early microglial response in experimental thiamine deficiency: an immunohistochemical analysis. Glia 1999; 25: 190–198. 9890633 10.1002/(sici)1098-1136(19990115)25:2<190::aid-glia9>3.0.co;2-b

