# Supplementary figures and images for: High-dose thiamine prevents brain lesions and prolongs survival of Slc19a3-deficient mice
Source: PLoS One. 2017 Jun 30;12(6):e0180279. doi: 10.1371/journal.pone.0180279 (PMC5493381; doi:10.1371/journal.pone.0180279)

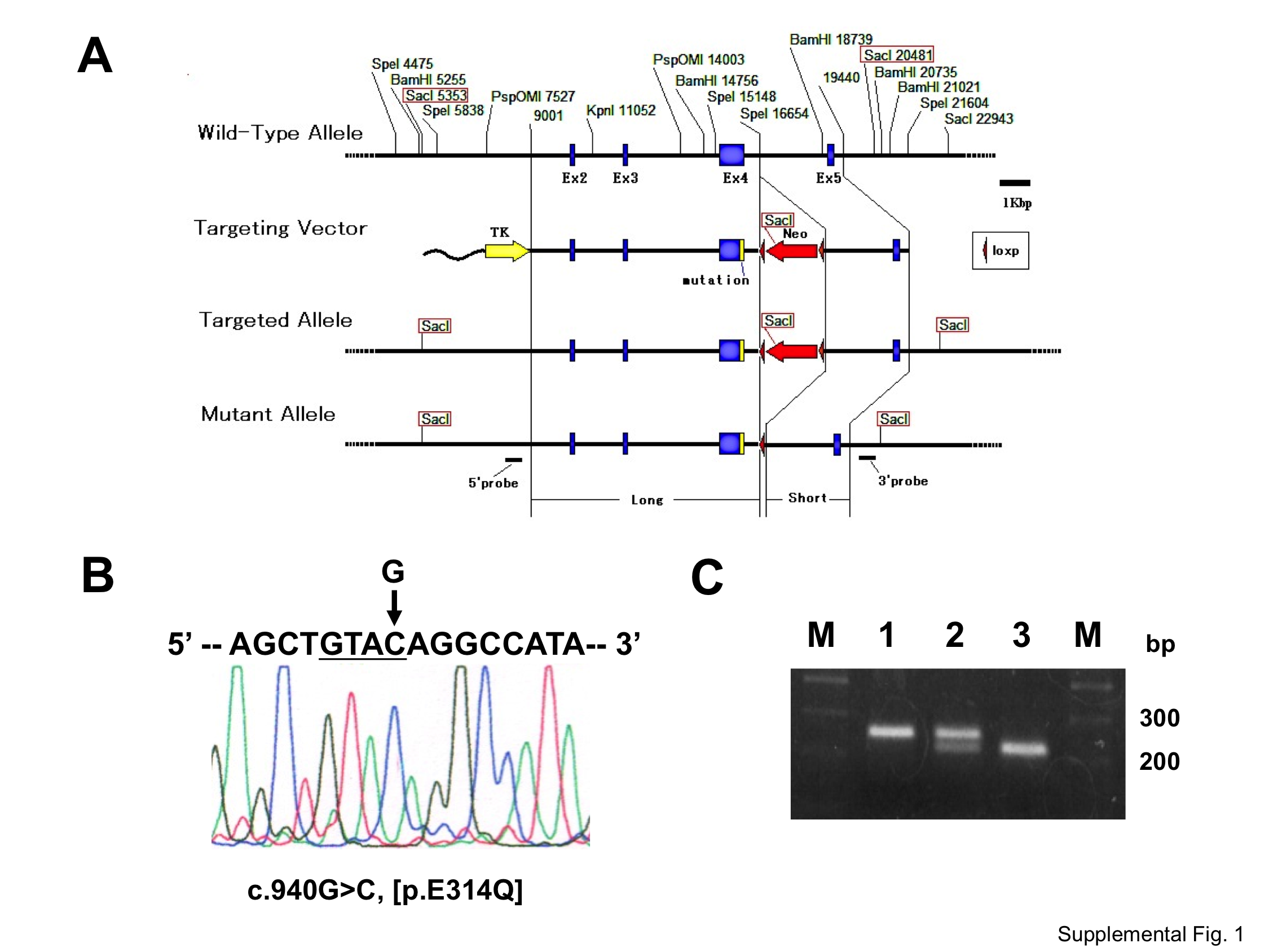

Supplement: S1 Fig — A. Schematic illustration of the construction strategy of Slc19a3 E314Q KI mice. The targeting vector contains a 3.0-kb 5' arm containing exon 3 with a neomycin selection cassette flanked by loxP sites and a 8.1-kb 3' arm containing exon 4–5 with the E314Q knock-in mutation (c.940G>C, [p.E314Q]) in exon 4. B. Confirmation of the genome sequence of homozygous E314Q KI mice. The mutation (c.940G>C, [p.E314Q]) in exon 4 was validated. The c.940G>C substitution generated a novel RsaI site (GTAC, underlined). C. Genotyping by PCR-RFLP analysis using RsaI. A primer pair (sense S1: 5′-agcaacccagatccagaaaat-3′; antisense A1: 5′-acacttacctccaaatgttgc-3′) was used to amplify a part of exon 4. RsaI was used to digest the 240-bp mutant (c.940G>C) PCR product, which generated the 209- and 31-bp PCR products. Lane 1, WT; lane 2, heterozygous E314Q KI mouse; lane 3, homozygous E314Q KI mouse. (TIF) [file pone.0180279.s001.tif]

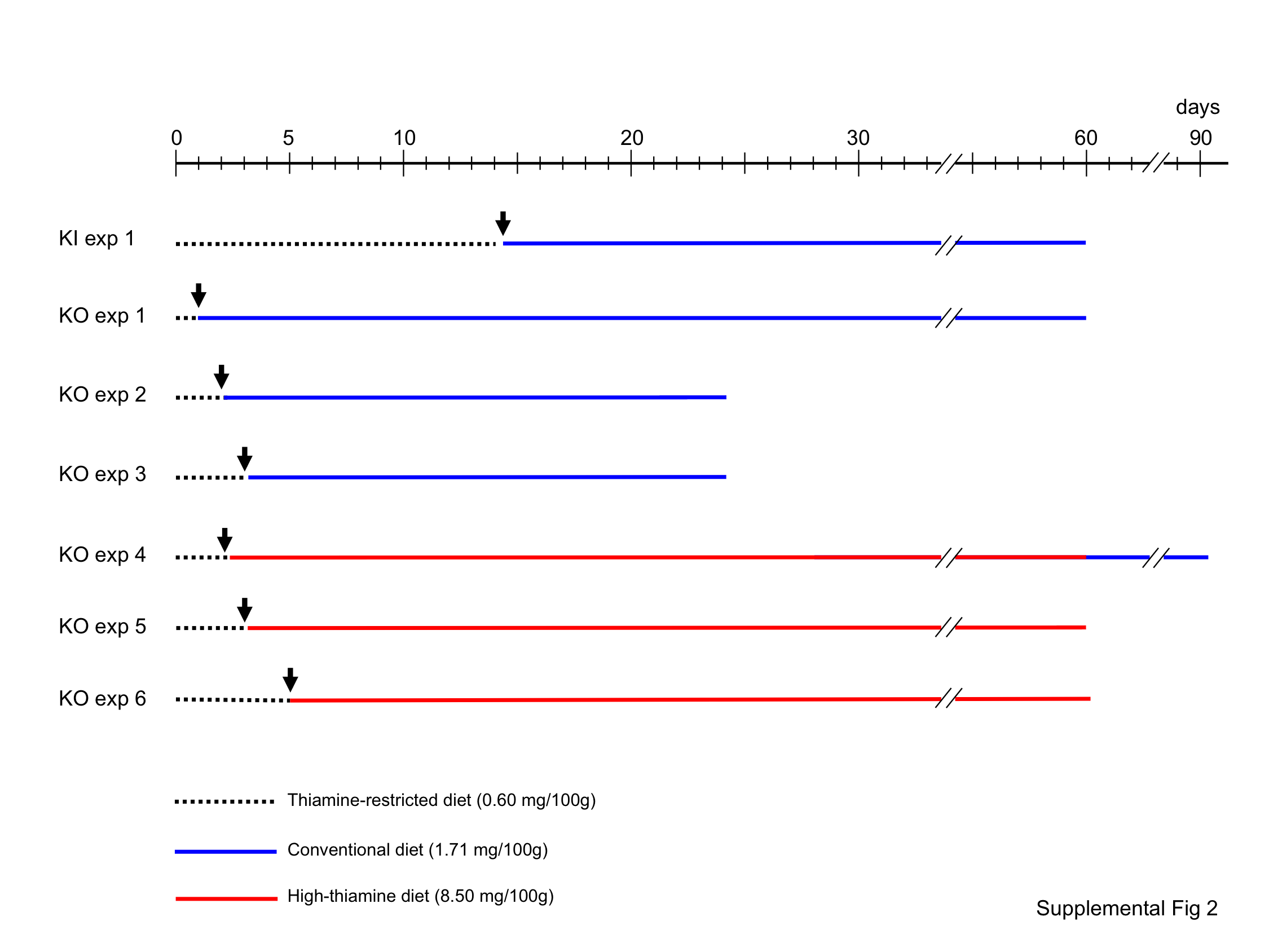

Supplement: S2 Fig — KI experiment 1: Homozygous KI mice aged 5–6 weeks were fed with a thiamine-restricted diet for 14 days and then changed to a conventional diet. KO experiments 1–3: Homozygous KO mice aged 5–6 weeks were fed with a thiamine-restricted diet for 1, 2, or 3 days and then changed to a conventional diet. KO experiments 4–6: Homozygous KO mice aged 5–6 weeks were fed with a thiamine-restricted diet for 2, 3, or 5 days and then changed to a high-thiamine diet. In KO experiment 4, recovered homozygous KO mice were reverted to a conventional diet at 60 days. (TIF) [file pone.0180279.s002.tif]

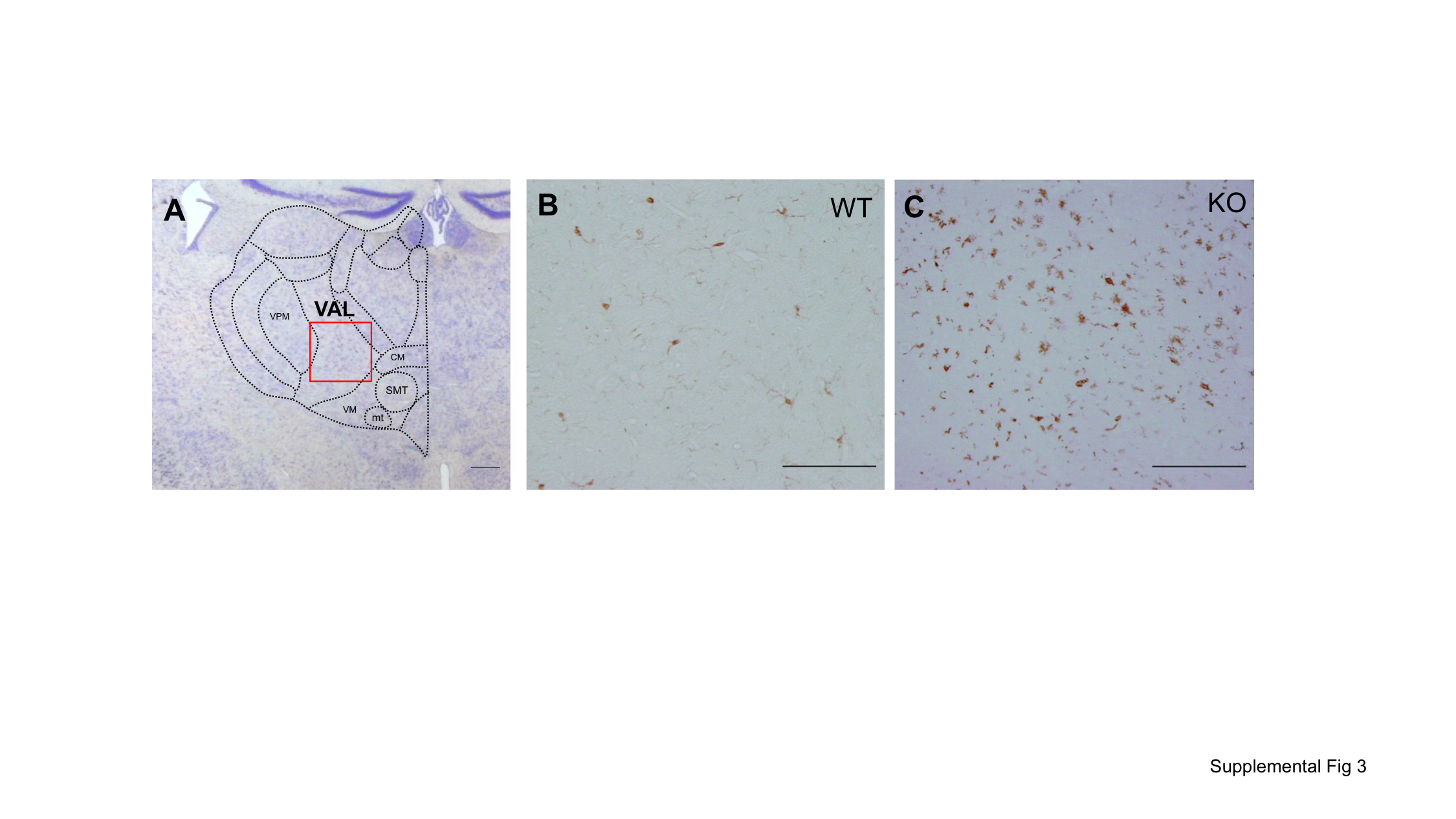

Supplement: S3 Fig — Iba1-immunopositive microglial cells in the VAL of WT (B) and homozygous KO mice (C) fed with thiamine-restricted diet for 8 days. Overview of the specific regions of the thalamic area of a WT mouse (A), with the red box indicating the region of VAL. Anti-Iba1 antibody (Wako Pure Chemical Industries, #019–19741, 1:500) was used. Scale bar, 200 μm. Note that homozygous KO mice (C) exhibit an increase in the number of activated microglia, which have larger cell bodies and thicker processes. (TIF) [file pone.0180279.s003.tif]
